# No Stress! Relax! Mechanisms Governing Growth and Shape in Plant Cells

**DOI:** 10.3390/ijms15035094

**Published:** 2014-03-21

**Authors:** Gea Guerriero, Jean-Francois Hausman, Giampiero Cai

**Affiliations:** 1 Department Environment and Agro-biotechnologies (EVA), Centre de Recherche Public—Gabriel Lippmann, 41 Rue du Brill, L-4422 Belvaux, Luxembourg; E-Mail: guerrier@lippmann.lu; 2 Dipartimento Scienze della Vita, Università di Siena, via Mattioli 4, I-53100 Siena, Italy

**Keywords:** plant cell growth, plant cell shape, expansion, turgor pressure, cytoskeleton, cell wall

## Abstract

The mechanisms through which plant cells control growth and shape are the result of the coordinated action of many events, notably cell wall stress relaxation and turgor-driven expansion. The scalar nature of turgor pressure would drive plant cells to assume spherical shapes; however, this is not the case, as plant cells show an amazing variety of morphologies. Plant cell walls are dynamic structures that can display alterations in matrix polysaccharide composition and concentration, which ultimately affect the wall deformation rate. The wide varieties of plant cell shapes, spanning from elongated cylinders (as pollen tubes) and jigsaw puzzle-like epidermal cells, to very long fibres and branched stellate leaf trichomes, can be understood if the underlying mechanisms regulating wall biosynthesis and cytoskeletal dynamics are addressed. This review aims at gathering the available knowledge on the fundamental mechanisms regulating expansion, growth and shape in plant cells by putting a special emphasis on the cell wall-cytoskeleton system continuum. In particular, we discuss from a molecular point of view the growth mechanisms characterizing cell types with strikingly different geometries and describe their relationship with primary walls. The purpose, here, is to provide the reader with a comprehensive overview of the multitude of events through which plant cells manage to expand and control their final shapes.

## 1. Introduction

The structural organization and functional specialization of plant tissues is achieved through metabolic specialization (an emblematic example is represented by plants with CAM (crassulacean acid metabolism)) and morphogenetic programs, which depend on the capacity of cells to expand and change their shape. The morphogenetic events regulating the 3D patterning of plant structures are a subject of great fascination not only for developmental biologists and for plant physiologists, but also for physicists and mathematicians, who are more and more interested in understanding the intrinsic laws determining the wide array of plant cell geometries, e.g., [1].

Since plant cells are not characterized by the ability of migrating during development (as animal cells), they achieve a perfect control over final shape by modulating cell division and expansion [2].

A key role in plant cell expansion is played by the cell wall: not anymore considered an inert stiff layer with the sole purpose of providing “armour” to the living protoplasts, the cell wall is a crucial cytological structure actively participating in several important physiological events [3–8].

The two most relevant parameters that control plant cell expansion are turgor pressure and mechanical properties of cell walls [9]. Plant cells are indeed subject to a noteworthy turgor pressure, which is due to a difference in solute concentration between the interior and the exterior. The pressure acts on the cell wall, which has to counteract this internal force and is therefore in a state of tensile stress [10]. However, when growth occurs, the wall has to allow extension by yielding to turgor pressure and, at the same time, new wall material has to be deposited to allow the re-formation of the wall mechanical resistance to pressure. The concepts of turgor pressure and cell wall mechanical properties were for the first time described in the seminal work by Lockhart [11], who elaborated an equation explaining the mathematics of plant cell expansion. The cells that expand have to overcome a barrier (the intrinsic wall yield threshold), beyond which plastic expansion takes place: this is represented in the Lockhart’s equation, where the coefficient of wall stiffness variation is taken into account. The turgor pressure and the physical and mechanical properties of the cell wall are factors that contribute to shape complex cellular geometries. In this respect, it is possible to understand the role of the cell wall during growth if one assumes that a plant cell regulates its volume by working as a hydrostat [12,13], where the cell wall acts as a sensor of the system. Whenever the concentration of the solutes in a plant cell increases (as for instance because of accumulation of photosynthates in a metabolically active cell), the turgor pressure increases and the wall experiences higher tensile stress; the subsequent response is a loosening of the cell wall which allows expansion of the cell. When the cell has expanded, a new equilibrium is reached, since the solutes are more diluted because of water inflow and the cell will strengthen the wall network to counteract the newly formed turgor pressure and to be able to initiate a new wave of expansion.

## 2. The Irreversible Expansion of Plant Cells

**Figure 1.**
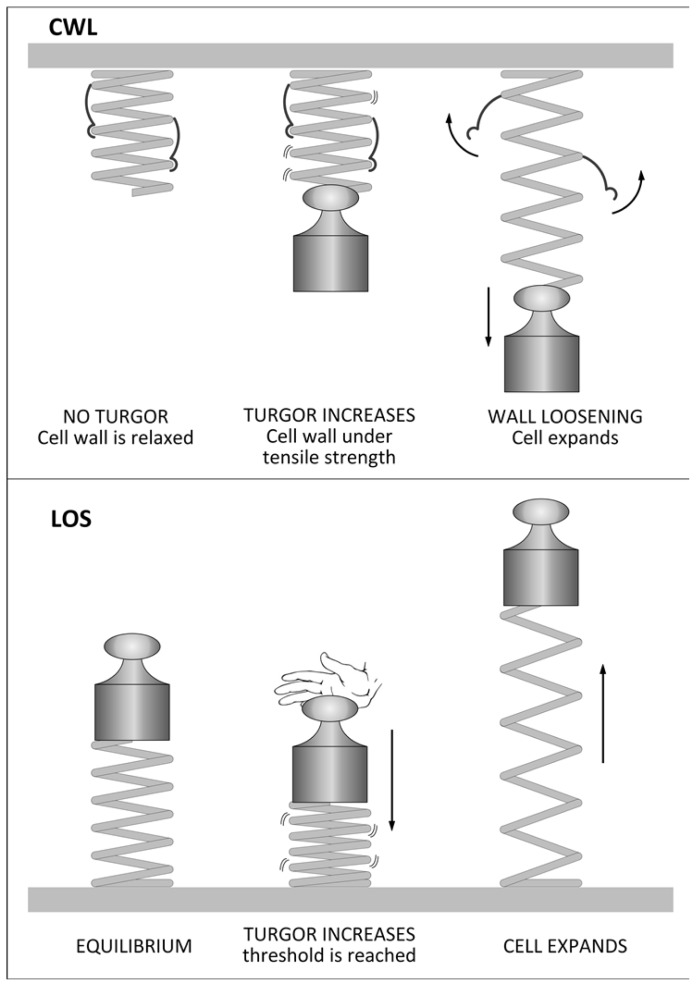
Schematic representation of the two principles (turgor pressure and wall tensile stress) regulating plant cell growth. The top part of the figure illustrates the CWL (Chemical Wall Loosening) mechanism by using a spring, hooks and a weight, while the lower part schematizes the LOS model. In the CWL model, the spring (which represents the cell wall), under the action of the weight (the internal turgor pressure), is in tensile stress and the potential energy of the system increases; when the transducer (wall loosening enzymes) releases the weight by unfastening the hooks holding the spring (which represent the inter/intra-chain bonds of wall polysaccharides), the energy accumulated is transformed into kinetic energy (growth). Therefore, the action of wall loosening enzymes is required for growth, according to the CWL model. According to the LOS model, the compression of the spring (*i.e.*, the cell wall) by an increase in turgor pressure (here represented as a hand pushing the weight), proceeds until a threshold is reached (wall yield threshold). Beyond this threshold, the wall yields and expansion occurs. Therefore, according to the LOS model, growth depends on the intrinsic properties of the wall.

**Figure 2.**
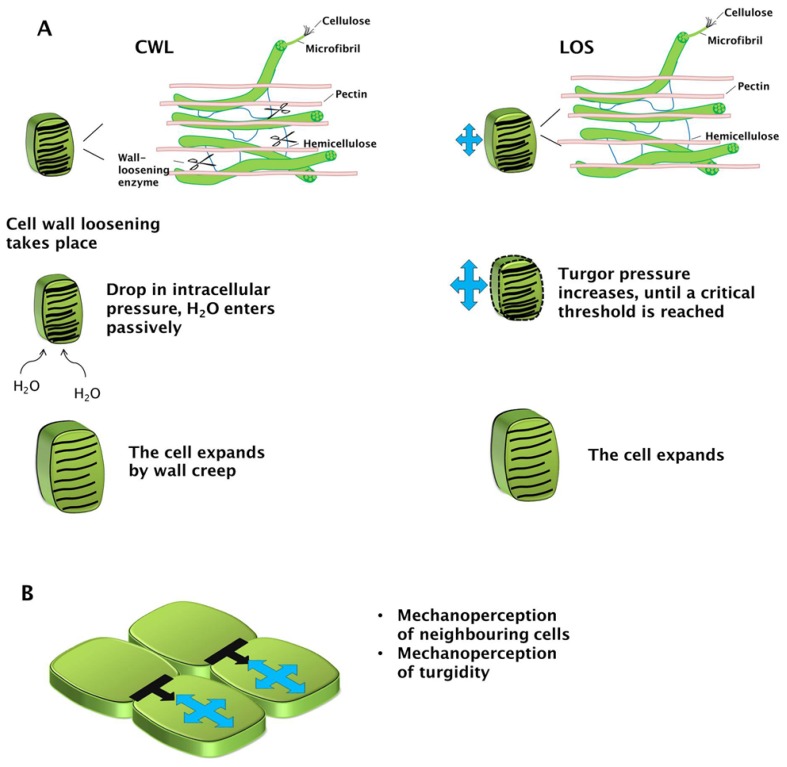
Models of plant cell expansion and mechanosensory signals. (**A**) Cartoons depicting the steps marking growth according to the CWL and LOS models; the dotted line represents the critical yield threshold of the wall; (**B**) Mechanosensory signals coming from neighbouring cells (black arrows) and from the turgidity of the expanding cells (blue arrows).

Plant cells can increase their cell volume by a reversible and an irreversible mechanism. The reversible mechanism is found in cells which undergo elastic deformation; the irreversible mechanism (which leads to growth) implies the generation of a mechano-hydraulic driving force which determines an increase of volume and therefore of surface area [10]. According to the classic Chemical Wall Loosening (CWL) model, this is favoured by a continuous decrease in wall stress [10]. A very simple principle in physics, *i.e.*, the transformation of energy via a transducer, can be invoked to explain the CWL irreversible expansion of plant cells. The cell wall can very simplistically be regarded as a spring held under tension by a weight, which represents the turgor pressure capable of extending the spring (the cell wall) and to determine an increase of the potential energy of the system (Figure 1). When enzymatic loosening of the wall occurs (the transducer), the irreversible conversion of the potential energy stored in the compressed spring will be transformed into kinetic energy (that is, in cell growth, Figure 1). Even if the spring undergoes an elastic expansion in the example, actually cell walls expand irreversibly (plastic expansion); however, the analogy can provide an easy visualization of the complex process that takes place *in vivo*. The CWL model of growth is indeed mediated via a process involving: (A) relaxation of cell wall tensile stress by primary and secondary wall loosening agents (*i.e.*, those causing or not stress relaxation directly; [2]); (B) decrease in turgor pressure (because of wall loosening which allows water influx); and (C) enlargement (by wall creep [10]; Figure 2).

Although the scientific community unanimously recognizes turgor pressure and wall relaxation as the two main factors controlling expansion, there is much discussion about which event is established first [14]. A theory was proposed about 10 years ago, called Loss of Stability (LOS) [15–17], according to which turgor pressure increases gradually until it reaches a critical threshold, beyond which the wall yields to the internal force (Figures 1 and 2). This yielding of the wall is dictated by the intrinsic material and geometrical properties of the wall itself. Irrespective of the model, turgor pressure and wall properties are key physical aspects underlying expansion.

Internal pressure and the vicinity of neighboring cells exert mechanical stimuli, which are sensed by the cell in expansion. Mechanosensing and mechanotransduction act on genes and affect plant morphogenesis [18]. A study of Hamant and co-workers [19] elucidated that induced stress at the tissue level (as for instance after cell ablation in *Arabidopsis* shoot apex, which triggers the formation of a void around the target cell) causes a re-orientation of the cortical microtubules parallel to the stress line. Microtubules mediate the mechanical stress signal and broadcast it to regulate morphogenesis. Therefore, mechanoperception is an important mechanism through which the expanding plant cell perceives the mechanical signals coming from the neighbours (Figure 2).

**Figure 3.**
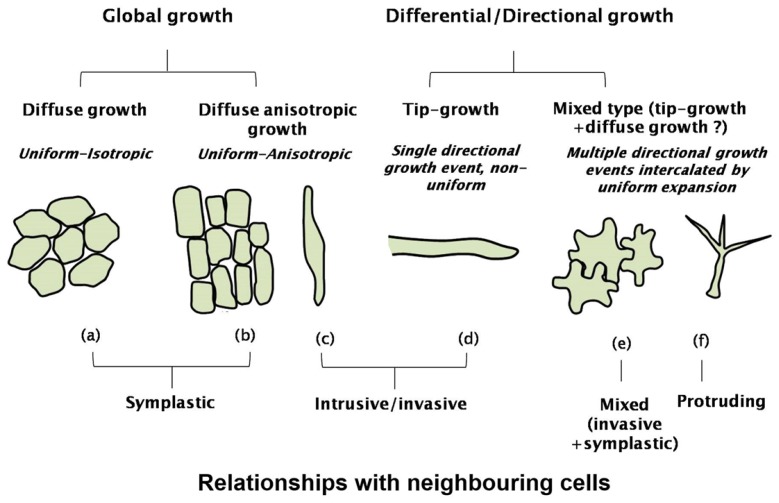
Cartoon depicting the two main plant cell growth events: global and differential/directional growth. Details concerning the cell types (**a**) fruit parenchyma cells; (**b**) shoot/root epidermal cells; (**c**) bast fibre; (**d**) pollen tube; (**e**) leaf pavement cells; (**f**) trichome and the relationship with the neighbouring cells are added to show the different expansion modalities displayed by plant cells. The question mark indicating the mixed-type growth refers to a hypothetical mechanism involving both tip- and diffuse growth.

Plant cells display two main mechanisms of growth: global and directional (or differential) [1]. The terms “diffuse” and “tip-growth” are widely used in the literature to designate the main growth modalities of plant cells, however we use here the terms “global” and “directional” as they better describe the details of the intervening mechanisms (namely uniform-isotropic/uniform-anisotropic). The global mechanism is found in fruit parenchyma cells, where uniform and isotropic expansion leads to an almost spherical shape and in epidermal cells of the shoot and roots where, however, expansion is anisotropic (Figure 3). Bast fibres and cells of the vascular tissue also show diffuse anisotropic expansion, which leads to long fusiform cells (Figure 3). Differential growth requires polarization of the cell, *i.e.*, the creation of areas of local growth. This event, which translates into the formation of a bulge, is the prelude to a series of other steps that are necessary for complex geometries, as jigsaw puzzle-like pavement cells or branched trichomes. Although the molecular details characterizing the growth of cells with such complex shapes have not been fully elucidated, it is plausible to assume that specific processes, not occurring in other cell types, are involved. Differential growth is accompanied by ultrastructural cytological differences.

Depending on how the cells expand with respect to the neighbours, three different mechanisms can be identified: symplastic, intrusive (or invasive) and protrusive growth [20]. During symplastic growth, the contact surfaces of the growing cells with the adjacent ones are kept constant; with intrusive growth, the elongating cells invade the middle lamellas of the neighbours; protruding growth takes place away from the plant surface (not within a tissue) and is thus not physically limited by any neighboring cells. Symplastic growth is typical of shoot/root epidermal cells and marks the initial phase of fibre development [20,21]; intrusive growth is found in bast fibres which can reach considerable lengths and is typical of polarized structures, such as pollen tubes; protruding growth is a hallmark of trichome formation [20].

## 3. Factors Affecting Plant Cell Growth

The deposition of cell wall necessarily requires that new materials are synthesized and incorporated into the cell wall during the extension process. The machinery (or even the same cell wall building elements) is transported within membranous structures that move actively along cytoskeletal elements. A strong body of evidence in the literature points indeed towards the existence in plants of a fine cross-talk between cell wall, cytoskeleton and endomembrane systems. This is quite intuitive if one considers how the intracellular trafficking of vesicles harbouring wall material is strictly dependent on the cytoskeleton, e.g., [22–26], and how cell wall biosynthesis and morphology can be affected when using drugs specifically altering cytoskeletal homeostasis and dynamics, e.g., [27,28].

Microtubules have been shown to play an important role in modulating plant cell growth; in particular, studies on the role of drugs selectively targeting microtubules have shown that their impairment causes a loss of growth direction with concomitant isotropic swelling [29]. The effect of microtubules on plant cell growth is not direct, since they do not directly stiffen the cell, but it is mediated via their guidance of cellulose microfibril deposition [30]. The orientation of the cellulosic microfibrils is indeed aligned with the underlying network of microtubules, whose tracks are followed by cellulose synthases (CESAs) [31]. Recently, mutations in *POM2/CSI1* (*CELLULOSE SYNTHASE INTERACTING 1*), which is required for CESAs movement along microtubules, were shown to affect elongation [32]. Many studies have described aberrant growth patterns in root or leaf epidermal cells of transgenic plants with altered expression patterns of tubulin or other genes related to microtubule dynamics/homeostasis [33]. An emblematic example is the macroscopic phenotype caused by helical twisting of plant organs: *lefty* mutants incorporating α-tubulins with missense mutations [33,34] and transgenic plants overexpressing a poplar microtubule-associated protein (PttMAP20) [35] show left-handed twisting. These are clear examples of the dramatic changes that it is possible to trigger when messing with microtubules, but many other genes are involved in determining a normal pattern of diffuse growth via microtubules. As further examples, we will mention here a few representative genes, as excellent reviews have already provided a broad description of the cytoskeleton-related genes affecting plant cells shape, e.g., [36]. Mutation in a group of genes called *PILZ* cause, in the mushroom-shaped embryos, the presence of a few enlarged cells [37,38]. In particular, these genes encode the mammalian orthologs of the tubulin folding cofactors (TFC) C (*PORCINO*), D (*CHAMPIGNON*), E (*PFIFFERLING*) and the G-protein Arl2 mediating the formation of α/β-tubulin heterodimers (*HALLIMASCH*) [37] and are required for the formation of microtubules array and vesicle trafficking during cytokinesis [38].

The gene *WAVE-DAMPENED 2* codes for a microtubule-associated protein whose high constitutive expression causes suppression of root waving, reduced anisotropic cell elongation, inverted handedness of hypocotyl and root twisting and whose suppression triggers reduced branching of trichomes [39].

Actin also plays an important role in diffuse growth: the localization of fine and bundles of F-actin modulates increased/decreased expansion and their random distribution (as for instance in the *DISTORTED* genes from *A. thaliana*) causes the formation of small misshapen cells [36,40].

A further recent study highlighted the role of actin in normal cell expansion: mutations in the *A. thaliana* villin genes *VLN2* and *3* (which promote actin bundling) showed twisting in the double mutant plants and in particular this defect was correlated to differences in cell lengths [41].

A very interesting study has recently allowed progress towards understanding the fine cellular dynamics regulating growth of plant cells: it was shown that plant cell growth is achieved through a response to the mechanical stress (generated by the adherence to adjacent cells) mediated via katanin, a protein involved in microtubule severing [42,43]. In particular, by studying symplastic growth in the shoot apical meristem (SAM) in a control and katanin mutant, the authors show that in normal conditions the cells respond to the mechanical forces by re-orienting microtubules [43]. In particular, katanin favours encountering of cortical microtubules and their organization in parallel arrays [43]. What also emerged from the study is that, while at the beginning mechanical stress triggers a more homogeneous growth, when it is increased, cells over-react and growth heterogeneity is enhanced [43]. This elegant study therefore highlights how complex the *in planta* mechanism regulating growth and morphogenesis can be.

Growth of plant cells is also controlled in a developmental manner, as specific genes are activated in the different stages. An emblematic example is represented by the stem, where different regions associated with specific cell growth stages can be identified. A recent study carried out on *A. thaliana* has allowed a rigorous monitoring of sequential stages during stem development through growth kinematics coupled to expression analysis [44]. The different stages analysed, namely elongation, maximum growth rate, growth cessation and fully matured stem, showed different gene expression profiles associated with the transition of the different growth phases [44]. Genes related to active protein synthesis (namely elongation factors) and cell-cell communication (e.g., LRR kinase and *CLE16*) are represented in the elongation phase. The maximum growth rate region shows genes involved in cell wall biosynthesis, namely a peroxidase (*PER64*) involved in lignification, arabinogalactan proteins (AGPs), *MYB61* transcription factor, xyloglucan endotransglycosylase/hydrolase (*MERI5B/XTH24*). The cessation of growth region shows upregulation in genes involved in secondary cell wall deposition, namely *CesA4*, *CesA7*, *CesA8*, those related to xylan biosynthesis and modification, e.g., *UDP-GLUCURONIC ACID DECARBOXYLASE 3* (*UXS3*), *IRREGULAR XYLEM 9*(*IRX9*), *FRAGILE FIBER 8*(*FRA8*), *XTH19*, *REDUCED WALL ACETYLATION1*(*RWA1*) [44].

The molecular studies addressing transcriptomics of different plant stem regions are powerful tools to investigate the role of specific genes in cell growth, cytoskeletal dynamics and cell wall metabolism. The availability of genome sequences for an ever-increasing number of species will enable researchers to extend these analyses to important non-model plants, as for instance fibre crops and therefore to deepen the understanding of the mechanisms involved in cell growth/expansion, cell wall biosynthesis and cytoskeleton dynamics.

## 4. A Special Mechanism of Global Growth: The Case of Bast Fibres

**Figure 4.**
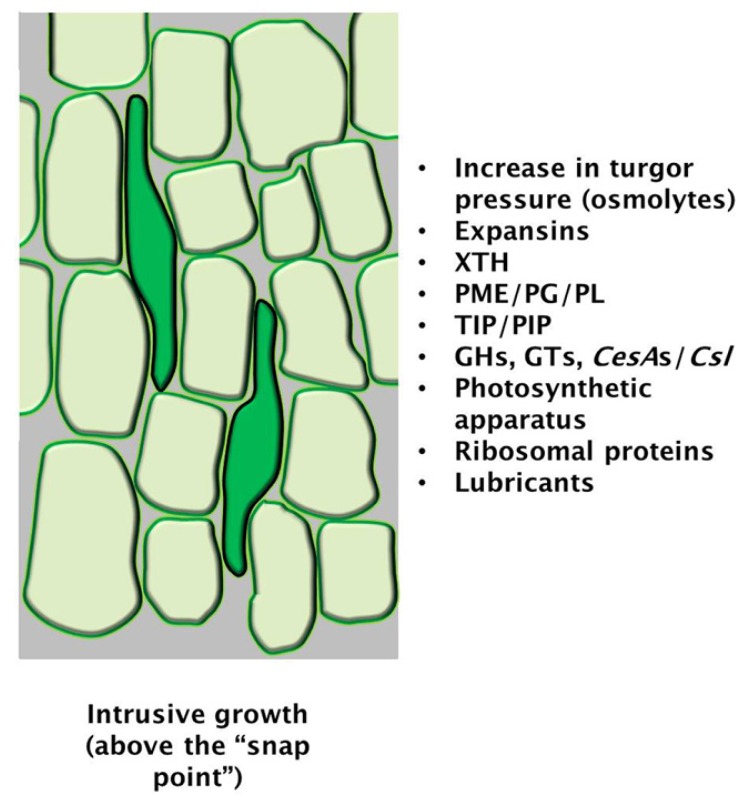
Cartoon representing the diffuse (intrusive) growth of bast fibres (dark green cell grows showing intrusive extremities, as in [20]). The box shows important factors/genes involved [20,47]. Abbreviations: XTH xyloglucan endotransglycosylase/hydrolase, PME pectin methylesterase, PG polygalacturonase, PL pectin lyase, TIP tonoplast intrinsic protein, PIP plasma membrane intrinsic protein, GHs glycosylhydrolases, GTs, glycosytransferases, *CesA*s cellulose synthases, *Csl* cellulose synthase-like.

Bast fibres, which are cellulose-rich fibres found in crops such as *Cannabis sativa* and *Linum usitatissimum*, are suitable models to study diffuse growth: it has indeed been shown that they display diffuse growth rather than tip-growth [45] and that the mechanism allowing them to reach considerable lengths against the impedance of the surrounding tissue is intrusive growth. Intrusive growth ensures that the number of fibres in a specific transverse section of the stem increases, while the total number of cells remains unchanged [20]. Intrusive growth involves penetration of the growing fibre extremities between the middle lamellas of adjacent cells: this requires that the surfaces of the fibres assume a pointy appearance (to help them “slide” between the neighbouring cells), which is achieved through the formation of “knees” [20] (Figure 4). The presence of these “knees” is a recognizable sign of active intrusive growth [20], a process involving uniform-anisotropic expansion [46].

Intrusive growth has been described as the analog of dendrite and axon growth in animals, and although cell migration is not a feature of the plant kingdom, this mechanism of elongation enables the actively growing plant cell to reach new regions [48]. As some of the fibres grow considerably in length, middle lamellas of numerous cells have to be detached [48]: nevertheless, no wound response is triggered by this elongation mechanism, as the plant clearly shows the ability of recognizing the self from the non-self (as, for instance, invading hyphae).

In order to achieve intrusive growth, the turgor pressure of the growing fibre must increase to promote extension: although osmolytes have never been measured in growing fibres, it is known that proteins involved in establishing a hydrodynamic equilibrium are actively expressed, namely aquaporins [20] (Figure 4). An important element during diffuse growth of bast fibres is the decrease of friction, which originates from the sliding of the expanding cell wall with that of its immediate neighbour. Lubrication of pectins in the middle lamella might play an important role in reducing friction forces [46].

Wall plasticity clearly plays a fundamental role in regulating diffuse growth: one of the wall-related genes shown to be involved in diffuse growth is pectin methylesterase (PME). Intermolecular links can form in the pectinaceous matrix of cells and one such link is that promoted by PME through contiguous de-esterification of methylesterified homogalacturonan (HG) and subsequent binding of calcium to the free carboxylic groups [47]. These bonds trigger the formation of the so-called “egg-box” structure, which confers stiffness to the wall. Such a stiff structure does not favour growth; therefore, it was proposed that PME negatively regulates intrusive growth [47].

Bast fibres elongate above the “snap-point” [21], a region of the shoot where the mechanical properties of fibre cells change significantly, while below it cell wall thickening occurs. This zonation allows molecular studies on the dynamics of cell wall biosynthesis occurring during the transition from active elongation to cessation of growth and subsequent thickening. As recently reviewed by Guerriero *et al*. [49], several studies have shed light on the expression of wall-related genes along the stem of mature fibre crops which, interestingly, mirror the stages of development along the hypocotyls (as shown for flax, [50]). In the elongation region, genes involved in primary cell wall biosynthesis, photosynthesis, hormone signaling are enriched, while those coding for AGPs, as well as β-galactosidases and glycosyhydrolases (GHs), were abundant at later stages of development [50].

## 5. Differential Growth to Generate Complex Geometries

The first event taking place in plant cells committed to complex shapes is the formation of a bulge, *i.e.*, an area of polarized growth. To redirect the plant cell shape towards a structure other than a sphere (because of turgor pressure, which is non-vectorial), the presence of two factors alone or in combination are necessary: anisotropy (dictated by the orientation of cellulose microfibrils) and non-uniform distribution of wall stiffness (generated by local wall softening) [36].

Wall-loosening enzymes, namely expansins, XTH and endo-1,4-β-d-glucanase, are agents which can trigger rapid modulations in the bonding of wall polysaccharides [51] and therefore create local mechanical alterations in the wall.

A bulge will create changes in the cytological endoarchitecture, as the plant cell perceives this event as the initiation of a local specialized area regulating growth direction: the appearance of actin patches is an event tightly linked to the onset of polar growth [36]. Actin polymerization is indeed responsible for the protrusion of membranes and for the motility of membranous vesicles within the polarized boundary regions [36]. The crucial role of actin is further demonstrated by mutations in the *Arabidopsis thaliana* orthologs of ACTIN-RELATED PROTEINS 2 and 3 (*WURM* and *DISTORTED1*) which cause a series of aberrant phenotypes in trichomes, pavement cells, hypocotyl cells and root hairs [52].

**Figure 5.**
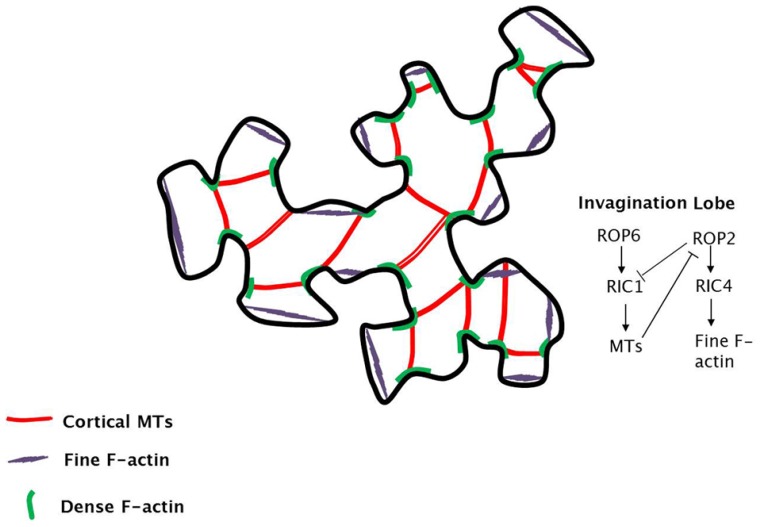
Schematic representation of the cytoskeletal architecture in a jigsaw puzzle-like pavement cell (as described in [36,53]). On the right, schematic molecular details on the relationship between Rho proteins and cytoskeleton are reported for the molecular control of interdigitated domains (invagination/lobe).

A regulated distribution of fine and dense filamentous actin (F-actin) mesh, together with microtubules, determines the further polarization of growth: areas with a fine actin mesh favour vesicle delivery, while those with a dense mesh constrain growth and act as a barrier to the movement of vesicles [36] (Figure 5). Microtubules provide support to the loosened cytoskeletal area and co-localize with zones showing dense actin mesh, thus further enhancing polarity [36] (Figure 5).

Recently, the role of Rho GTPases from plants (ROPs) has been surveyed [53] and it was recognized that this class of proteins plays a relevant role in breaking the symmetry of plant cells [54,55]. ROPs induce a self-organizing signalling, which controls the organization of complex cell structures, like the interdigitated cells of plant epidermis [53,54]. Lobe regions are enriched in fine F-actin and poor in microtubules, while invaginated areas are rich in microtubule arrays and dense F-actin [36] (Figure 5). The interdigitated architecture is finely regulated by the interplay of ROPs. As shown in Figure 5, in lobe regions, ROP2 is locally activated and it sequentially activates RIC4, which induces fine cortical F-actin accumulation; in invaginated regions, ROP6 is activated and induces the activation of RIC1, which favours the formation of microtubule arrays [53,54]. RIC1 negatively regulates ROP2 and microtubules suppress ROP2 activation [53,54] (Figure 5).

Bulging exerts also effects on the distribution of apoplastic proteins, through a passive mechanism which responds to the altered cell surface curvature. Very recently, the distribution of a GPI-linked lipid transfer protein (LTPG) has been studied in young and mature epidermal cells of *A. thaliana* and it was shown that the cellular distribution of this protein is dynamic and strictly dependent on the geometry of the cell and the wall curvature [56]. In particular, the LTPG would help seal the junctions surrounding adjacent cells, by promoting cuticular wax deposition and cell wall fortification [56].

## 6. Tip Growth

Certain plant cells possess a mechanism of apical growth that allows them to extend at a specific point along a directional axis. In this way, these cells can penetrate through other tissues (such as in the case of pollen tubes) or through the soil (as in the case of root hairs). The tip growth mechanism is exemplified by the pollen tube, a critical cell in the process of fertilization in higher plants (angiosperms and gymnosperms) [57]. This cell must penetrate the tissues of the female reproductive apparatus in order to reach the ovary and then the ovules. To achieve this purpose, pollen tubes (as well as root hairs) must have an elongated shape, capable of penetrating tissues. In these cells, the growth process is restricted and limited only to the cell apex where a set of different molecules works in concert to ensure that the growth process takes place correctly. The growth mechanism is most likely autonomous and self-maintained; however, as the pollen tube must reach a specific point of the ovary, its growth direction must be controlled by the female reproductive apparatus [58] (not discussed in this review). Tip growth necessarily requires the shape of pollen tubes to be constantly maintained throughout the elongation process [59,60]. In many cases, the process of pollen tube growth is defined as “pulsed growth” because the pollen tube alternates between phases of slow and fast growth. It is supposed that this process underlies the real mechanism that governs the growth of pollen tubes, *i.e.*, the presence of several sub-circuits that oscillate between two different states [61].

The mechanism of tip growth in pollen tube requires considerable accumulation of secretory vesicles at the tube apex, the so-called “clear zone” [62]. Vesicles are vectorially transported along actin filaments through myosins associated with their surface [63]. While bundles of actin filaments direct vesicles towards the apical area, the so-called “actin fringe” (a kind of band/disk of short actin filaments whose organization oscillates in phase with the growth of pollen tubes [64]) is likely to filter the membrane materials that reach the apical area and/or to focus secretory vesicles in the apex. In addition to the above-mentioned components, secretory vesicles should also deliver the enzymes necessary for cell wall modification, such as PME [65] and transglutaminase (TGase) [66]. The specific site of vesicle fusion in the apical plasma membrane is unclear and has been questioned [67]. Pectins and PME, as a well as CESA, are likely secreted in the tip domain [68] while callose synthase (CALS), apart from being inserted in the apical plasma membrane, is also likely delivered and inserted in distal regions of the pollen tube [22,69].

Pectins are secreted in the methyl-esterified form thus forming the initial (soft) cell wall, whose visco-elastic nature is fundamental for cell elongation because it might be stretched by the internal turgor pressure [70]. Modification (de-esterification) of pectins in the cell wall is carried out by PME, which is secreted in a form inactivated by a specific protein inhibitor (PME inhibitor or PMEI) [71]. Activation of PME necessarily requires the removal of PMEI [65], which critically might control the correct assembly of cell wall. Activation of PME also requires changes in proton concentration [72].

The secretion of methyl-esterified pectins and PMEI determines the degree of visco-elasticity of the apical cell wall (the secretion of new material in the apical cell wall is visually exemplified by the increase in thickening [61]). As soon as the apical cell wall of pollen tubes becomes sufficiently visco-elastic, turgor pressure exerts a stretching activity. While secretion represents the slow phase of growth, distension of the apical region as exerted by turgor pressure represents the fast phase of growth. In recent years, this specific point has been debated. Some researchers have argued that turgor pressure is not constant in the pollen tube but its variation is the engine that drives the actual growth of pollen tubes (the so-called hydrodynamic model) [73]. According to other researchers, the lack of experimental data in support of this hypothesis invalidates the theory of turgor variation and puts the basis for a different theory based on the visco-elasticity of the apical cell wall that, when above a certain threshold, allows the pollen tube to be stretched, while turgor pressure remains almost unchanged [74]. In addition to temporal differences in the construction of the pollen tube cell wall, spatial aspects may also have some relevance. In this context, it was suggested that the difference in apparent stiffness between the apex and flanks of pollen tubes could be explained by the different geometry existing between the apical and subapical regions. The spatial-based theoretical model predicts that the difference in stiffness of the cell wall does not require modifications in the rigidity, but these differences in cell wall structure must nevertheless exist to allow pollen tubes to grow. It is also important that the theoretical model is validated experimentally to confirm predictions [75]. This set of data indicates that a comprehensive model of the mechanism of pollen tube growth is not yet developed.

Assuming that turgor pressure does not change substantially during the growth phase [75], such pressure exerts different effects depending on the type of cell wall. After secretion, new methyl-esterified pectins can soften the texture of the apical cell wall by mixing with esterified pectins (slow phase of growth). The decrease in stiffness and the simultaneous increase of the visco-elastic nature of the apical cell wall allows the turgor pressure to locally stretch the pollen tube cell wall thereby deforming its shape (fast phase of growth) [9,76,77]. It is very likely that the stretching process activates a cascade of events, including the entry of Ca^2+^ (via stretched-activated Ca^2+^ channels) and the resulting depolymerization of actin filaments in the subapical region (these aspects will not be treated in this review) [78–80]. Consequently, PME (and its inhibitor PMEI), CESA and CALS are secreted at the apex but in different functional states. PME is probably blocked by its inhibitor PMEI, which prevents the formation of esterified (acid) pectins within secretory vesicles [65]. As soon as the inhibitory effect of PMEI stops, methyl-esterified pectins are converted into acid pectins thereby stiffening the cell wall. Hypothetically, the stages of secretion, slow growth, cell wall softening, fast growth by turgor-mediated extension, conversion of pectins in the acid form, and strengthening of the cell wall can be seen in the typical production of “rings” of acid pectins in pollen tubes exhibiting pulsed growth [81]. The oscillation in the pollen tube growth has often been the subject of modeling in which the deposition and the turnover of new cell wall material facilitates the mechanical deformation. In this way, it is theoretically possible to reproduce the morphology of the pollen tube, its growth oscillations, and the relationship between cell wall thickness, cell morphology and growth rate [82]. At the same time, the differences in cell wall stiffness guarantee that turgor pressure exerts its stretching activity specifically at the tube apex so as not to deform the overall pollen tube structure.

If the secretion of methyl-esterified pectins and their progressive transformation into de-esterified (acid) pectins, coupled to turgor pressure, is the engine capable of driving pollen tube growth, other elements have to structure the distal cell wall in order to stabilize the cylindrical shape of pollen tubes allowing them to grow in the extracellular matrix of the female reproductive apparatus. Cellulose and callose are deposited after the first 15–20 μm from the tube apex and they stabilize the cell wall albeit with different roles [83,84]. Callose is much more abundant than cellulose and it could represent a critical step in the evolution of angiosperms [85,86]. The deposition of the callose layer strengthens the cell wall while the deposition of callose plugs in distal regions most probably contributes to the maintenance of turgor pressure. Callose is synthesized by CALS, which is localized in the plasma membrane of pollen tubes [87] and is probably secreted in an inactive form at the pollen tube apex [88]. Actin filaments appear to play an essential role in directing CALS-containing vesicles to the tube apex, while microtubules might be involved in the accumulation of CALS in distal regions of pollen tubes in relation to the deposition of callose plugs [22]. The deposition of callose in pollen tubes is probably not homogeneous, but it may follow a “scaled” trend reminiscent of acid pectins in tubes with pulsed growth [89]. Callose is a non-crystalline polymer and most likely it does not contribute to stabilize the directional growth as it is consistently deposited starting from the sub-apex, then relatively distant from the area where growth direction is established. Its main function is thus probably linked to the maintenance of the finger-like shape of pollen tubes and to the resistance against turgor pressure.

**Figure 6.**
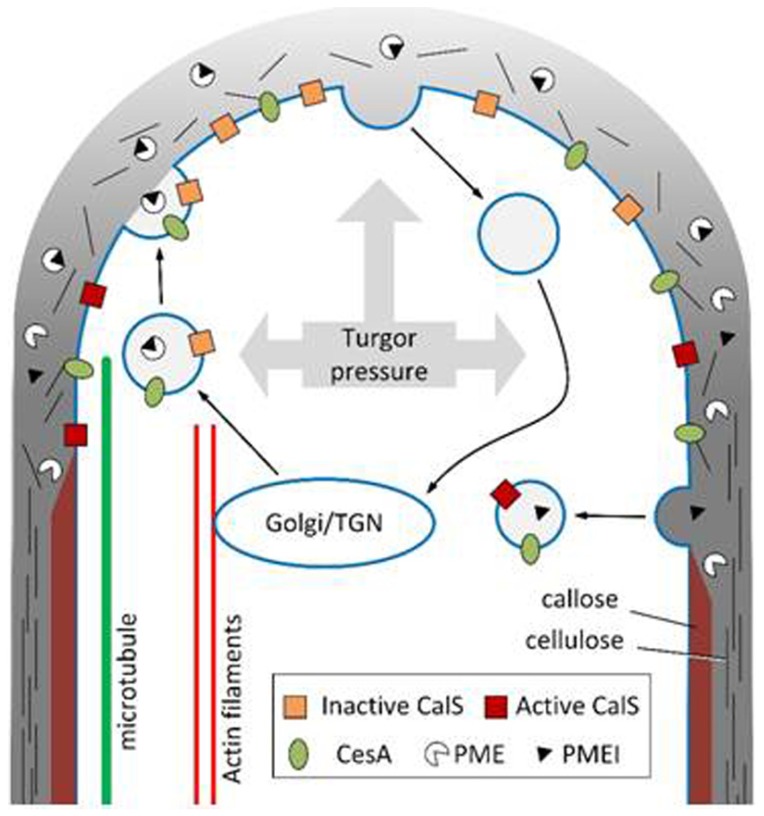
Schematic diagram of the secretion process in pollen tubes. Only the key elements involved in cell wall synthesis are shown (PME, PMEI, CESA, CALS). The gradient of gray in the cell wall indicates the status of methyl-esterification of pectins (light gray: fully methyl-esterified pectins; dark gray: acid pectins). Cellulose is shown as random black lines in the tube apex and as more organized lines in distal parts. Callose is shown as red bands in the pollen tube shanks. TGN, Trans-Golgi-Network. Actin filaments (red lines) are shown as interacting with the Golgi, indicating that they are involved in the transport of vesicles to the apex. Microtubules (green lines) are shown as positioned in the cortical region where they presumably could interact with CALS and CESA. Secretion is indicated to occur not exactly at the apex but in a region immediately close. Endocytotic processes are hypothesized to occur both at the extreme apex and in more distal regions [67,99].

The structural stability of pollen tubes is also probably maintained by the deposition of a second crystalline polymer (cellulose). Hypothetically, the abundant use of callose to withstand and counteract turgor pressure could have led to a decrease in cellulose levels as they are observed in the angiosperm pollen tube [90]. Cellulose of pollen tubes is hypothetically synthesized by CESA proteins or, more likely, by cellulose synthase-like proteins (CSL) [69]. Some CSL genes have been identified as important in the growth of pollen tubes [91]. Using either heterologous antibodies or fluorescence-tagged constructs, the secretion site of CESA was located at the tube apex [22,69]. Given that cellulose is a crystalline polymer, it may also have a directional function. However, the stabilization of growth direction is unlikely to require the deposition of additional polymers. Although structurally different, callose and cellulose are more likely contributing to stabilize the shape of pollen tubes. Nevertheless, the function of cellulose is still enigmatic. Cellulose is probably produced at the apex of pollen tubes but in a disorganized way and therefore unable to contribute to growth directionality [22,69]. In subapical regions, cellulose microfibrils are likely more organized and help therefore stabilizing the final shape of pollen tubes. In support of this hypothesis, inhibitors of cellulose biosynthesis (for example 2,6-dichlorobenzonitrile) can cause abnormal growth with rupture of pollen tubes and uneven deposition of the cell wall [92]. Furthermore, the proper expression of a gene encoding a cellulose synthase-like (CSLA) is essential for the growth of *Arabidopsis* pollen tubes [91]. Two other genes coding for different cellulose synthase-like (CSLD1 and CSLD4) have proved as important for the correct texture of the cell wall in *Arabidopsis* pollen tubes [69]. Therefore, even if present at low levels in the cell wall, cellulose seems essential for the proper growth of pollen tubes. We do not know the molecules controlling the organization of cellulose microfibrils in pollen tubes, but microtubules may intriguingly play this role. In somatic cells, a precise relationship exists between microtubules and deposition of cellulose microfibrils [93]. First, microtubules are necessary for the correct insertion of CESA in the plasma membrane through intermediate compartments [94,95]. Secondly, microtubules are used to direct the movement of CESA in the plane of the plasma membrane, at least in the first steps of cellulose synthesis [31]. We ignore whether this interaction also exists in the pollen tube, but microtubules (whose role is still unclear) may be important for the directional growth of pollen tubes (“directionality” is a generic term, but the data currently available do not allow using more specific terms) [96]. However, it should be mentioned that the depolymerization of microtubules by specific inhibitors does not cause apparent damages to pollen tube growth [97], apart from modifications of the pulsed growth (but this may not be directly related to alterations in the deposition of cellulose) [98]. A cartoon summarizing the events involved in pollen tube tip growth is depicted in Figure 6.

## 7. Conclusions and Future Perspectives

The rich variety of plant tissues is achieved through the specialization of cell types, which determine morphogenesis, organogenesis and therefore the overall physiology. This specialization is guaranteed by the “moldability” of plant cells, which are able to assume specific shapes, by activating cellular programs that remodel the cell wall and the underlying cytoskeletal network. The pivotal role played by the cell wall-cytoskeleton continuum is indeed obvious when one considers plant cell growth; we have here seen how physiologically and morphologically relevant alterations of this continuum can be. Additional important information on growth and shape determination can be obtained in the future by studying the mechanosensory function of the cell wall-cytoskeleton system and its broadcasting mechanism in the expanding plant cell.
